# Neural Processes Underlying Mirror-Induced Visual Illusion: An Activation Likelihood Estimation Meta-Analysis

**DOI:** 10.3389/fnhum.2020.00276

**Published:** 2020-07-31

**Authors:** Umar Muhammad Bello, Georg S. Kranz, Stanley John Winser, Chetwyn C. H. Chan

**Affiliations:** ^1^Department of Rehabilitation Sciences, The Hong Kong Polytechnic University, Hong Kong, China; ^2^Department of Physiotherapy, Yobe State University Teaching Hospital, Damaturu, Nigeria; ^3^Department of Psychiatry and Psychotherapy, Medical University of Vienna, Vienna, Austria; ^4^Applied Cognitive Neuroscience Laboratory, The Hong Kong Polytechnic University, Hong Kong, China; ^5^University Research Facility in Behavioral and Systems Neuroscience, The Hong Kong Polytechnic University, Hong Kong, China

**Keywords:** mirror-induced visual illusion, activation likelihood estimation, meta-analysis, cuneus, premotor

## Abstract

**Introduction:** Neuroimaging studies on neural processes associated with mirror-induced visual illusion (MVI) are growing in number. Previous systematic reviews on these studies used qualitative approaches.

**Objective:** The present study conducted activation likelihood estimation (ALE) meta-analysis to locate the brain areas for unfolding the neural processes associated with the MVI.

**Method:** We searched the CINAHL, MEDLINE, Scopus, and PubMed databases and identified eight studies (with 14 experiments) that met the inclusion criteria.

**Results:** Contrasting with a rest condition, strong convergence in the bilateral primary and premotor areas and the inferior parietal lobule suggested top-down motor planning and execution. In addition, convergence was identified in the ipsilateral precuneus, cerebellum, superior frontal gyrus, and superior parietal lobule, clusters corresponding to the static hidden hand indicating self-processing operations, somatosensory processing, and motor control. When contrasting with an active movement condition, additional substantial convergence was revealed in visual-related areas, such as the ipsilateral cuneus, fusiform gyrus, middle occipital gyrus (visual area V2) and lingual gyrus, which mediate basic visual processing.

**Conclusions:** To the best of our knowledge, the current meta-analysis is the first to reveal the visualization, mental rehearsal and motor-related processes underpinning the MVI and offers theoretical support on using MVI as a clinical intervention for post-stroke patients.

## Introduction

A plane mirror provides an instant visual feedback of body appearance and posture, thereby influencing self-awareness and aiding in complex visually guided tasks (Jenkinson and Preston, 2017). A plane mirror inverts the reflected image; e.g., the left limb appears as the right when positioned at the midsagittal plane (Bähr et al., 2018). The visual feedback created by the illusion of the left-to-right limb has been adopted as the basis of mirror therapy for patients with neurological disorders, e.g., stroke survivors (Matthys et al., 2009).

Mirror-induced visual illusion (MVI) was first introduced in late 1990s by Ramachandran to alleviate phantom limb pain in patients (Ramachandran and Rogers-Ramachandran, 1996). MVI was later adopted as clinical intervention for treating hemiparesis due to stroke (Altschuler et al., 1999). During mirror therapy, a patient places the paretic hand behind the mirror, whereas the unaffected hand stays in front of the reflecting surface of the mirror (Guerraz, 2015). The mirror is placed in an erect position corresponding to the body midline of the patient such that the paretic upper limb is hidden from the view (Guerraz, 2015). Common MVI protocols involve the unaffected hand engaging in different movements whilst the patient looks into the mirror and observes the movements as if they are performed by the paretic hand hidden behind the mirror. MVI was found to facilitate the motor recovery of paretic limbs amongst stroke survivors (Rosén and Lundborg, 2005). Evidence gathered from clinical reviews support the effectiveness of MVI on improving the functional regains of the upper and lower limbs (Broderick et al., 2018; Thieme et al., 2018; Zeng et al., 2018).

Deconinck et al. (2015) and Arya (2016) qualitatively collated the results of neuroimaging studies on neural substrates that mediate MVI. Deconinck et al. (2015) observed that MVI appeared to facilitate activities in the motor network despite the findings being largely inconsistent. The MVI effects were related to the increase in attention control via “increased cognitive penetration” (Deconinck et al., 2015). Deconinck et al. (2015) explained that the inconsistent results may be due to the small sample sizes and methodological variations across the studies reviewed. Supplementing Deconinck et al.'s study, Arya (2016) concluded that MVI facilitated the ipsilesional primary motor cortex (M1) via a top-down influence on the ipsilesional premotor cortex, resulting in the augmentation of neuroplasticity in the affected hemisphere amongst stroke survivors. Both papers reported widespread mirror-induced activations of the fronto-temporo-parietal, occipital and cerebellar brain regions. Findings from qualitative analyses would have the advantage of collating the results of studies and might align with the interests of researchers. However, researchers would face the challenge of whether the observations are robust and occur more than by chance. The present study attempted to use a meta-analytic method to test MVI hypotheses by pooling the activations of neural substrates found in functional brain imaging studies on MVI. The results offer evidence on the possible effects and underlying neural processes of MVI and hence increase our understanding on its potential role in the motor recovery of post-stroke patients.

Activation likelihood estimation (ALE) is a coordinate-based statistical method for consolidating the foci of the neural substrate(s) being activated when subjects engage in a task; such method is used across individual studies that report experiment(s) (Eickhoff et al., 2009, 2012; Turkeltaub et al., 2012). Previous ALE meta-analyses were conducted to collate and locate the neural substrates associated with motor imagery (Hétu et al., 2013) and action observation (Caspers et al., 2010). Furthermore, a recent ALE meta-analysis approach was used to identify the neural substrates associated with movement execution, motor imagery and action observation (Hardwick et al., 2018).

The current study aimed to use the ALE method to collate and identify the neural substrates that sub-serve the MVI processes in healthy adults and to examine how the sides of the hand (i.e., left vs. right) would modulate brain activations during task manipulation. Studies that recruited post-stroke patients were not included because of the inadequate number of studies and the limitations against the standards set by the ALE method. Understanding the effects of MVI on healthy adults contributes to knowledge on neural mechanisms and is essential to understanding the effect of treatment on post-stroke patients under the influence of brain lesions and functional abnormalities. In this study, we conceptualized MVI is to visualize moving hand images over-imposing on the “static” hand, thereby producing visual illusions. These motor-related visualizations result in top-down sensorimotor planning, execution and control processes. We hypothesized that in contrast to a condition involving movement without mirror visual feedback, the visualization in MVI would yield a significant convergence of activations in visual and motor-associated areas. Moreover, in contrast to a resting or control condition, the MVI effect would yield activations in the motor-associated network, particularly the premotor cortex, M1 and cerebellum, in line with increased top-down motor facilitation and activations in the precuneus and inferior parietal lobule ipsilateral to the moved hand.

## Methods

### Study Selection

The search for eligible studies for inclusion was conducted in accordance with the preferred reporting items for systematic review and meta-analysis protocol (PRISMA-P) (Moher et al., 2015). The search covered functional brain neuroimaging studies on MVI in the CINAHL, MEDLINE, Scopus, and PubMed databases from inception until November 2019. No restriction was set on the year of publication. The search terms were constructed under two themes: mirror therapy and neuroimaging modalities. Related and similar terms for each theme were developed, and the search used Boolean “OR” for individual terms and Boolean “AND” to combine the terms of the two themes. The search terms under mirror therapy included “mirror therapy,” “mirror visual illusion,” and “mirror illusion,” whereas those under neuroimaging modalities included “functional magnetic resonance imaging,” “positron emission tomography,” “fMRI,” and “PET.” Additional articles were manually searched from the reference lists of the included articles and existing systematic reviews.

The title, abstract and full text of each study were obtained in accordance with the method described above. The inclusion criteria were as follows: (1) test protocol on the instant effect of MVI involving the active movement of the right or left hand whilst observing mirror images superimposed on the opposite static hidden hand, (2) healthy adults (18 years or above) as subjects, (3) whole-brain group analysis results of functional magnetic resonance imaging or positron emission topography, (4) inclusion of a minimum of five participants, and (5) mapping of brain coordinates using Montreal Neurological Institute (MNI) or Talairach and Tournoux space.

### Data Extraction

The activation coordinates, number of experiments and sample size of each experiment were extracted and organized in accordance with the guideline provided by GingerALE 3.0.2 (available at: http://brainmap.org). Brain coordinates reported in the Talairach and Tournoux reference space were converted to the MNI reference space using the brain coordinate conversion options in GingerALE software (Lancaster et al., 2007).

### Activation Likelihood Estimation (ALE) Method

The analyses were performed in accordance with a recent coordinate-based meta-analysis guideline (Müller et al., 2018). ALE software version 3.0.2 (Eickhoff et al., 2009, 2012; Turkeltaub et al., 2012) was used to conduct all the data analyses, which began with generated modeled activation maps by pooling the task-related activations at the voxel level in the foci identified across the experiments (Turkeltaub et al., 2012). The ALE scores were yielded by pooling all the activation maps (Hardwick et al., 2018). Cluster-level FWE thresholding was used to guide the meta-analysis because it has greater sensitivity and specificity and is less prone to type-1 error in terms of convergence in comparison with voxel-wise thresholding (Eickhoff et al., 2016). The brain coordinates from experiments involving the same group of subjects were pooled into one experiment to control for sample overlap (Turkeltaub et al., 2012; Müller et al., 2018). Statistical significance was set at a corrected threshold of *p* < 0.05 (threshold permutation at 1000 cluster-forming threshold at a voxel level of *p* < 0.05) (Zheng et al., 2019). The foci labeling which showed significant pooled convergence used the probabilistic cytoarchitectonic maps of human brain in the SPM Anatomy Toolbox v2.1 (Eickhoff et al., 2005). MRIcro software with an MNI template (www.mricro.com) was used to visualize the results of the meta-analyses.

### Data Analyses

The overall meta-analysis entails pooling the activation foci from all the experiments involving the active movement of either the right or left hand in the MVI paradigm. To isolate the MVI effect on one side of the brain, flipping of activation coordinates was performed as previously reported (Witteman et al., 2012; Favre et al., 2014). We flipped the activation coordinates generated by experiments in which the right hand was actively moved to isolate the MVI effect on the left hemisphere. This process involved multiplying the x-coordinates of the foci yielded on the basis of right hand movements by “−1” (Witteman et al., 2012). The coordinates generated by the active movement of the right hand were flipped in association with the generation of reliable findings in motor-associated areas and on the basis of interhemispheric discrepancies in the brain mask size according to the ALE method (Eickhoff et al., 2005).

To examine the effect of the actively moving hand on brain activations, we conducted separate analyses by pooling the activation coordinates generated during the active movement of the right or left hand (without flipping the coordinates).

Activation foci were also pooled separately for studies that conducted second-level analysis by contrasting the MVI effect against active hand movement without mirror visual feedback as a control condition.

## Results

### Number and Description of Included Articles

**Figure 1:**
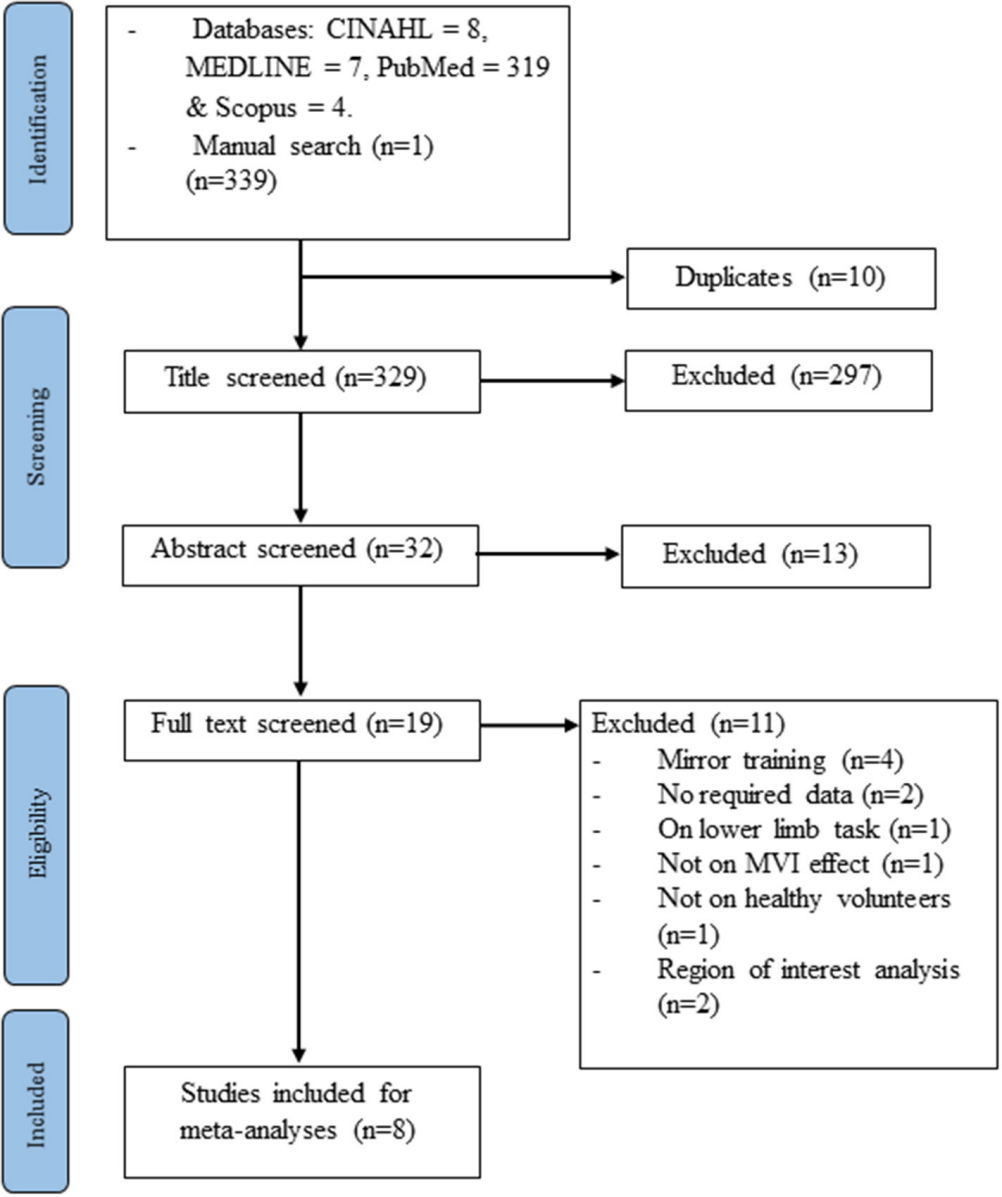
Study flowchart.

The electronic and manual search yielded 339 studies. After the removal of 10 duplicate records and the retrieval of the full information, another 321 studies were excluded. Eight studies (Dohle et al., 2004; Matthys et al., 2009; Numata et al., 2013; Wang et al., 2013a; Fritzsch et al., 2014; Diers et al., 2015; Milde et al., 2015; Manuweera et al., 2018) that reported experiments met the inclusion criteria (see Figure 1 and Table 1). All the studies tested the effect of MVI experiments involving upper limb movements. A total of 14 experiments reported 182 foci amongst 127 healthy subjects. Four studies (Dohle et al., 2004; Matthys et al., 2009; Numata et al., 2013; Wang et al., 2013a) reported results of contrast analysis between the MVI effect against a baseline/resting condition and a control condition (involving hand movement without mirror visual feedback). Three studies (Fritzsch et al., 2014; Diers et al., 2015; Milde et al., 2015) reported results of contrast analysis between the MVI effect and a baseline/resting condition, whereas a single study reported the result of contrast analysis between the MVI effect and a control condition (Manuweera et al., 2018). Nine experiments in seven studies (Dohle et al., 2004; Matthys et al., 2009; Numata et al., 2013; Wang et al., 2013a; Fritzsch et al., 2014; Diers et al., 2015; Milde et al., 2015) required the participants to engage in active movements of the right hand in the MVI paradigm, whereas four experiments in four studies involved the active movement of the left hand (Dohle et al., 2004; Numata et al., 2013; Wang et al., 2013a; Fritzsch et al., 2014) (Table 2).

**Table 1:** Summary of the included studies for the meta-analysis.

**References**	**Subjects characteristic**	**Task/mirror setup**	**No. of exp**.	**Coordinate space**	**Contrast(s)**	**Sentence 1: instruction during assessment Sentence 2: no. of moving hand(s) displayed**
Diers et al. (2015)	20 Healthy participants Mean age: 31.26 ± 7.74 M/F ratio: 5/15	Lightly open and close right fist at rate of 1 Hz/VR mirror box and conventional mirror box	2	MNI	MVI > static	To focus on inverted hand image. VR mirror box: 1; Conventional mirror box: 2.
Dohle et al. (2004)	6 Healthy participants Mean age: 29.0 ± 1.5 M/F ratio: 2/4	Finger-thumb opposition movement at frequency of 1 Hz/real-time video recording projected on to LCD screen	2	Tal	MVI > static MVI > control	To fixate on the hand on the screen. Both experiments: 1.
Fritzsch et al. (2014)	15 Healthy participants Mean age: 33.7 (22–56) M/F ratio: 9/6	Finger-thumb opposition movement/real-time video recording projected onto an MRI-goggle	2	MNI	MVI > static	To focus on inverted hand image. Both experiments: 1.
Manuweera et al. (2018)	20 Healthy participants Mean age: 25.6 ± 3.9 M/F ratio: 12/8	Finger flexion toward a target/real time video recording	1	MNI	MVI > control	To focus on inverted hand image. 1.
Matthys et al. (2009)	18 Healthy participants Mean age: 28.5 (22–48) M/F ratio: 10/8	Finger tapping with the right hand at ~1.5 Hz/mirror box	1	MNI	MVI > rest MVI > control	To focus visually on the mirror reflection. 2.
Milde et al. (2015)	20 Healthy participants Mean age: 31.3 ± 7.7 M/F ratio: 5/15	Repeated closing and opening of right hand at a frequency of 1 Hz/mirror glass and mirror box	2	MNI	MVI > baseline	To focus on visual reflection of inverted hand image. Mirror glass: 1; mirror box: 2.
Numata et al. (2013)	13 Healthy participants Mean age: 23.5 (20–32) M/F ratio: 6/7	Finger-thumb opposition task/mirror box	2	MNI	MVI > rest MVI > control	To observe moving hand reflected on the mirror. 1.
Wang et al. (2013a)	15 Healthy participants Mean age: 33.7 (22–56) M/F ratio: 9/6	Opposition movement sequence of the index finger and thumb/images projected to the participants online via LCD goggles	2	MNI	MVI > static MVI > control	To focus on inverted hand image. 1.

*MVI, mirror-induced visual illusion; MNI, Montreal neurological institute; Tal, Talairach and Tournoux; LCD, liquid crystal display; NR, not reported. FMRI was used in all the included studies and all the recruited subjects were right-handers*.

**Table 2:** Summary of ALE meta-analysis and sub-analyses.

**Meta-analyses**	**Number of experiments**	**Number of foci**
Overall meta-analysis (right–left flipped coordinates in experiments with active movement of the right hand)	13	152
Active movement of left hand in MVI paradigm	4	48
Active movement of right hand in MVI paradigm	9	104
Activation coordinates generated from contrast analysis between the MVI effect and active hand movement without mirror visual feedback (right–left flipped coordinates in experiments with active movement of the right hand)	–	30

Eight studies on MVI involving post-stroke patients were identified (Merians et al., 2009; Michielsen et al., 2011a,b; Bhasin et al., 2012; Wang et al., 2013b, 2017; Saleh et al., 2014, 2017; Novaes et al., 2018). Amongst them, majority (Merians et al., 2009; Bhasin et al., 2012; Wang et al., 2013b, 2017; Novaes et al., 2018) reported findings based on the region of interest method and do not satisfy the inclusion criteria (Müller et al., 2018).

### Overall Meta-Analysis

**Figure 2:**
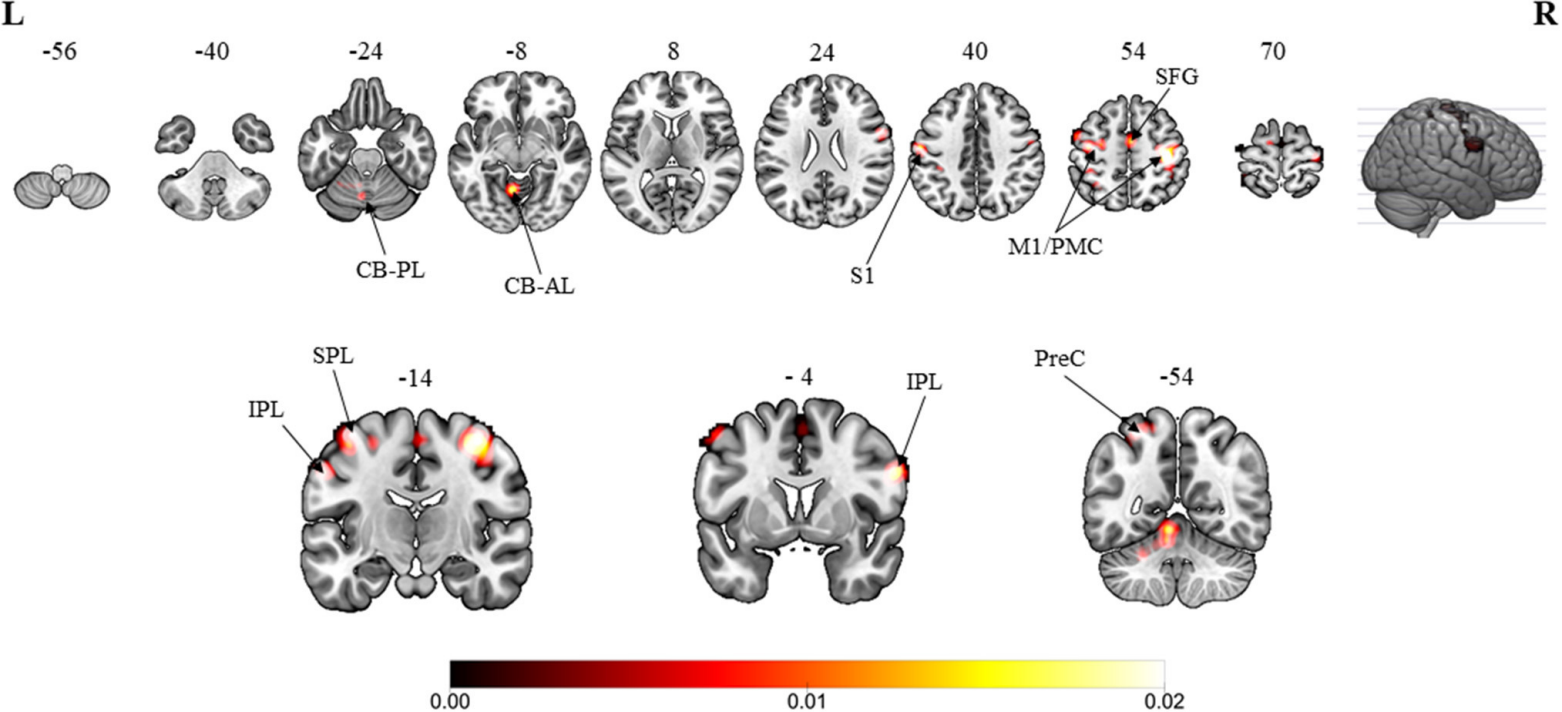
Overall meta-analytic results showing convergence of brain areas found to associate with the MVI condition when contrasted with the baseline/resting condition (13 experiments with 152 foci). Note: The MVI tasks involved moving left hand (right-left flipped coordinates for moving right hand). Labels: CB-PL, cerebellum-posterior lobe; CB-AL, cerebellum-anterior lobe; S1, primary somatosensory cortex; M1/PMC, primary motor cortex/premotor cortex; SFG, superior frontal gyrus; IPL, inferior parietal lobulem; SPL, superior parietal lobule; PreC, precuneus.

The overall results involved the contrast of pooled brain activations elicited in the MVI moving hand condition, i.e., observing the mirror on which images of the movements superimposed on the hand (static) hidden behind the mirror in contrast to those elicited under the baseline/resting condition (without movement of both hands). Consistent convergence was revealed in the bilateral M1 (Figure 2, *Z*-score: contralateral to moving hand = 7.63; ipsilateral to moving hand = 5.15), the premotor cortex (6.12; 4.34) and the inferior parietal lobule (3.32; 3.45) clusters. In terms of the *Z*-score, the strength of convergence in the primary and premotor cortices were stronger in the hemisphere contralateral to the moving hand in comparison with that in the ipsilateral hemisphere. Regions that were consistently found in the hemisphere ipsilateral to the moving hand were in the primary somatosensory cortex (*Z*-score = 4.70), superior frontal gyrus (3.61), superior parietal lobule (4.10), precuneus (2.98), cerebellum-anterior lobe (5.60) and the cerebellum-posterior lobe (3.26). The brain coordinates, *Z*-scores and their ALE values can be found in Appendix A.

### MVI Moving Left Hand (vs. Baseline/Resting Condition)

The moving left hand MVI protocols involved active movements of the left hand. Significantly strong convergence was found in the bilateral M1, premotor cortex and inferior parietal lobule. Regions that were consistently found specific to the ipsilateral (i.e., left side) hemisphere included the superior frontal gyrus, medial frontal gyrus, superior parietal lobule and precuneus. By contrast, regions specific to the contralateral (i.e., right side) hemisphere included the primary somatosensory cortex and insula cortex (Table 3).

**Table 3:** Convergent brain clusters found associated with moving left hand in the MVI paradigm (4 experiments with 48 foci).

**Cluster**	**Anatomical location**	**Side**	**BA**	***Z*-score**	**ALE value**	**MNI Coordinates value**		
						***X***	***Y***	***Z***
1	Primary somatosensory cortex	R	3	4.73	0.012	38	-26	54
	Primary motor cortex	R	4	4.57	0.012	42	-14	50
	Inferior parietal lobule	R	40	4.04	0.009	38	-36	58
2	Primary motor cortex	L	4	5.55	0.015	-40	-10	54
	Superior frontal gyrus	L	6	4.37	0.011	-12	-6	66
	Premotor cortex	L	6	3.52	0.007	-50	2	54
	Medial frontal gyrus	L	6	2.72	0.005	-4	-6	60
3	Superior parietal lobule	L	7	4.90	0.013	-30	-52	58
	Inferior parietal lobule	L	40	3.82	0.008	-32	-44	62
	Precuneus	L	7	3.72	0.008	-16	-52	62
4	Premotor cortex	R	6	4.80	0.013	56	4	26
	Insula cortex	R	13	2.70	0.005	46	2	8

*ALE, Activation Likelihood Estimation; BA, Brodmann's Area; MNI, Montreal neurological institute*.

### MVI Moving Right Hand (vs. Baseline/Resting Condition)

In comparison with the moving-left-hand paradigm, the moving of the right hand resulted in convergence (i.e., left side) in the contralateral M1 and premotor cortex clusters. Specific to the ipsilateral hemisphere (i.e., left side) brain areas were the transverse temporal gyrus, supramarginal gyrus, superior temporal gyrus and insula cortex (Table 4).

**Table 4:** Convergent brain clusters found associated with moving right hand in the MVI paradigm (9 experiments with 104 foci).

**Cluster**	**Anatomical location**	**Side**	**BA**	***Z*-score**	**ALE value**	**MNI Coordinates value**		
						***X***	***Y***	***Z***
1	Primary motor cortex	L	4	7.84	0.033	-38	-20	58
	Premotor cortex	L	6	3.93	0.012	-60	6	30
2	Transverse temporal gyrus	R	41	4.39	0.014	62	-16	8
	Supramarginal gyrus	R	40	4.05	0.012	64	-18	18
	Superior temporal gyrus	R	22	3.72	0.011	68	-34	18
	Insula cortex	R	13	3.32	0.008	52	8	0

*ALE, Activation Likelihood Estimation; BA, Brodmann's Area; MNI, Montreal neurological institute*.

### Other MVI Analysis (vs. Active Hand Movement Without Mirror Visual Feedback)

**Figure 3:**
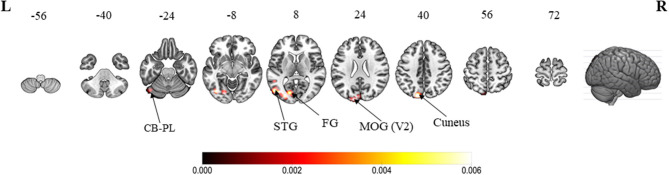
ALE meta-analytic results showing convergent brain clusters as a result of contrast between MVI condition with active hand movement without mirror visual feedback (30 foci). Note: The MVI tasks involved movements of the left hand (right–left flipped coordinates for right hand movements). Labels: CB-PL, cerebellum-posterior lobe; STG, superior temporal gyrus; FG, fusiform gyrus; MOG, middle occipital gyrus.

When compared with active hand movement without mirror visual feedback, the convergent clusters associated with the MVI processes were in the ipsilateral cuneus (*Z*-score = 4.69), lingual gyrus (4.50), middle occipital gyrus (4.16), superior temporal (fusiform) gyrus (2.87), precuneus (2.87), and posterior lobe of the cerebellum (2.87) (Figure 3 and Appendix B).

## Discussion

### Overall Meta-Analysis

This study attempts to explore the cluster convergence induced by the MVI to reveal its possible involvement in visualization and motor-related processes. The first major finding is that the MVI showed strong convergence in the bilateral M1, premotor cortex and inferior parietal lobule. The bilateral hemispheric convergence, particularly those in the ipsilateral hemisphere, may have been contaminated by the activations associated with the movements of the “moved” hand. In the ipsilateral hemisphere, our findings show convergence in the cuneus, lingual gyrus, middle occipital gyrus, superior temporal (fusiform) gyrus, precuneus, and the posterior lobe of the cerebellum.

The premotor cortex plays a major role in decoding visuo-motor movements (Schluter et al., 2001) and sensory feedback (Rushworth et al., 1998; Hoshi and Tanji, 2007). Together with the M1, it mediates movement execution (Hoshi and Tanji, 2007). The premotor cortex also mediates fine motor coordination (Hardwick et al., 2018), and its activation is considered the precursor for MVI-related ipsilateral M1 activation (Hamzei et al., 2012). In contrast to a baseline/resting condition, the convergent results of the clusters in bilateral M1 and premotor cortex are consistent with those reported in existing reviews on the MVI effect (Deconinck et al., 2015; Arya, 2016). The contrasts with the contralateral “moved” hand condition in this study demonstrate that the ipsilateral M1 and premotor cortex are likely not involved in the MVI processes. This finding offers further evidence to support the notion that the results reported in previous studies may be due to interhemispheric transcallosal transfer (Tinazzi and Zanette, 1998) and top-down sensorimotor facilitation via attention control (Deconinck et al., 2015). Future studies should collect further evidence on confirming these confounds.

Using transcranial magnetic stimulation, other studies have revealed that MVI results in increases in the amplitude of motor-evoked potentials in the hemisphere ipsilateral to the “moved” hand; a marker of M1 activity amongst healthy volunteers (Garry et al., 2005; Fukumura et al., 2007) and stroke survivors (Kang et al., 2011, 2012). MVI also results in increases in the amplitudes of the lateralized readiness potential and event-related desynchronization in the ipsilateral M1 regions (Lee et al., 2015; Debnath and Franz, 2016). Some MVI studies do not report significant ipsilateral M1 involvement (Funase et al., 2007; Mehnert et al., 2013). These inconsistent results may be due to the variation in the content of mirror images (i.e., the speed, complexity, and clarity of movement images); mirror therapy setups and task designs could further serve as sources of heterogeneity in mirror therapy research.

The second major finding in this study is that MVI shows specific convergence in the hemisphere ipsilateral to the “moved” hand in the precuneus, cerebellum (anterior and posterior lobe), primary somatosensory cortex, superior frontal gyrus, and superior parietal lobule. Amongst these clusters, the precuneus and superior parietal lobule seem to play a key role in linking the visualization process of the mirrored images of the “moved” hand (see below) to motor-related planning, execution, and control processes (primary somatosensory cortex, superior frontal gyrus, and cerebellum). The precuneus has been identified as a hub for coordinating cognitive and motor-related processes (Zigmond et al., 2014). These processes include recollection and memory, self-processing operations, retrieval of spatial information during motor imagery, body image representations and visuospatial perception (Ogiso et al., 2000; Cavanna and Trimble, 2006; Zigmond et al., 2014; Deconinck et al., 2015). In this case, a crucial step in MVI is to include the processing of the mirrored images of the “moved” hand in the form of visuospatial hand information, such as finger and wrist movements (Wolbers et al., 2003). It is also plausible that the gestures of the “moved” hand could be perceived as motor programming of the “static” hand (Eng et al., 2007). Such propositions are further supported by the convergences revealed in the supramarginal gyrus and the insular cortex from the contrast between MVI (moving right hand) and the baseline/resting condition. Our finding on the involvement of the precuneus in the MVI is consistent with studies on stroke survivors (Wang et al., 2013b; Saleh et al., 2014, 2017). Different studies have reported the association of activations in the precuneus with viewing mirror-inverted images of one's own “moved” limb (Dohle et al., 2011; Mehnert et al., 2013).

### Other MVI Analysis

Four visual-related convergent clusters worth mentioning are the ipsilateral cuneus, lingual gyrus, fusiform gyrus and middle occipital gyrus (MOG) which were revealed in the contrast between the MVI and “moved” hand without mirror visual feedback conditions. Both the cuneus and MOG are involved in the early visual processing of object orientation, motion, form and color (Gegenfurtner et al., 1996; Vanni et al., 2001). The lingual and fusiform gyri mediate the object color attribute judgement task (Wang X. et al., 2013). Meanwhile, the fusiform gyrus is associated with shape and color information processing (Simmons et al., 2007; Yang et al., 2015). Taken together, these results suggest that visualizing the mirrored images of the “moved” hand would have begun rather early (MOG) and been perceived as external to the body (i.e., cuneus) intensively, whereas the images would have been rich in context (i.e., lingual and fusiform gyri Table 5).

**Table 5:** Summary of neural substrates showing convergent clustering and their associated mental processes.

**Neural substrate**	**Associated functions**
Primary motor cortex (M1)	Motor planning and execution (Chouinard and Paus, 2006; Tarkka and Hautasaari, 2019)
Premotor cortex	Motor coordination (Hoshi and Tanji, 2007; Hardwick et al., 2018)
Inferior parietal lobule	Sense of agency, self-other discrimination (Chaminade and Decety, 2002; Uddin et al., 2006)
Primary somatosensory cortex	Sensory processing (kinesthesia) (Naito et al., 2007)
Superior frontal gyrus	Working memory, sensorimotor processing (Boisgueheneuc et al., 2006; Li et al., 2013)
Precuneus	Self-processing operations and visuo-spatial processing (Ogiso et al., 2000; Cavanna and Trimble, 2006; Deconinck et al., 2015)
Superior parietal lobule	Spatial localization of body part (Felician et al., 2004)
Cerebellum	Motor control and kinesthesia (Grill et al., 1994; Pisotta and Molinari, 2014)
Cuneus	Basic visual processing (Vanni et al., 2001)
Middle occipital gyrus	Basic visual processing and illusory or subjective contours (Gegenfurtner et al., 1996)
Fusiform gyrus	Color and shape processing (Simmons et al., 2007; Yang et al., 2015)
Lingual gyrus	Color knowledge processing (Wang X. et al., 2013)
Insula cortex	Sense of agency (Farrer and Frith, 2002)

### Motor Imagery and MVI Processes

Two major theories that have been adopted by researchers for describing the MVI phenomenon are mirror neuron system theory (Matthys et al., 2009; Hamzei et al., 2012) and motor imagery (Stevens and Stoykov, 2003; Fukumura et al., 2007). The results of this study, particularly those in the ipsilateral (to the “moved” hand) convergent clusters, support the latter theory more than the former. Results of an earlier meta-analytic study on the mirror properties of mirror neuron system suggested substantially larger involvements of brain areas including posterior inferior frontal gyrus, inferior parietal lobule, superior parietal lobule, middle temporal gyrus, dorsal and ventral premotor cortices, and the cerebellum (Molenberghs et al., 2012). Earlier studies associated mirror-liked properties with neural activities in the premotor cortex, superior temporal gyrus, middle temporal gyrus, and inferior frontal gyrus (Rizzolatti et al., 1996; Strafella and Paus, 2000). The limited overlaps in the premotor cortex between these studies and the present study suggests that the mental processes sub-serving the MVI are likely beyond the mirror neuron system. This proposition concurs with the observation made by Deconinck et al. (2015). Mental imagery theory stipulates that the rehearsal of the motor images throughout the imagery processes involves activations in motor-related cortices, as well as in the precuneus, superior and inferior parietal lobules, insula cortex, dorsolateral prefrontal cortex, putamen, and cerebellum (Hanakawa et al., 2003; Kuhtz-Buschbeck et al., 2003; Fourkas et al., 2006; Higuchi et al., 2007; Guillot et al., 2009; Glover and Baran, 2017). These neural substrates largely overlap with the ipsilateral clusters, i.e., primary somatosensory cortex, premotor cortex, precuneus, cerebellum, superior, and inferior parietal lobules and insula cortex, found in this study. The overlaps in these neural substrates suggest other major mental processes when engaging in MVI. They include access to sensorimotor representation, maintenance, and transformation of visuo-motor images, and motor preparation (Hétu et al., 2013; Simos et al., 2017). With similar widespread neural networks (Hétu et al., 2013; Hardwick et al., 2018), motor imagery could serve as the theoretical underpinning for MVI processes. In addition, the involvement of the primary somatosensory cortex in MVI is an indication of the increased ipsilateral sensory processing of the observed hand movement. This finding could explain the integration of kinesthetic sensation associated with the hidden static hand when subjects engage with the mirror therapy setup (Diers et al., 2010; Hadoush et al., 2013; Chancel et al., 2016).

### Comparing the Active Movement of the Right or Left Hand in the MVI Paradigm

The convergence of activations in the bilateral M1 and premotor cortices was only found in the left- but not in the right-hand movement condition. The left hand was the non-dominant hand. By contrast, the right-hand-movement condition yielded convergence in the contralateral hemisphere. Two studies using magnetoencephalography revealed higher involvement of the ipsilateral M1 in the left-hand-movement condition (Tominaga et al., 2009; Hadoush et al., 2013). These results seem to suggest that left hand movements may elicit stronger activations in the ipsilateral M1 in comparison with those of the right hand. Hadoush et al. (2013) speculated that the stronger ipsilateral activations may be attributed to the mirror-inverted right-hand image (more dominant) generated during left hand (less dominant) movements. Future studies should address the effect of hand dominance in MVI.

### Implications of the Findings

The revealed convergence clusters explain the effect of MVI. Observing hand movements results in increasing activations in the clusters in the motor-related system, i.e., M1 and premotor cortex. The motor-related effects appear to be mediated by a series of additional cognitive processes, resulting in activations in clusters in the parietal and visual regions. These clusters suggest the heavy involvement of visuo-motor imagery processes in MVI, including the maintenance, visualization and transformation of somatosensory and motor images. However, different opinions on the theoretical underpinning which accounts for the MVI effect exist. Our findings provide quantitative neural substrates and the mental processes which may sub-serve the MVI effect. Understanding these processes is important for the design of MVI protocols when conducting future experiments and providing clinical intervention for post-stroke patients. Subjects or patients should receive clear instructions to ensure that proper visuo-motor imagery process is engaged throughout the protocol. In addition, conducting the protocol in a quiet and appropriately illuminated environment can also enhance the quality of the images for processing.

### Strength and Limitations of Our Study

The present ALE meta-analysis provides quantitative neural substrates that modulate the MVI processes. Through subsequent sub-analyses, we reported the effects of hand dominance on the neural activity in MVI. To control for the sample overlap, we combined brain coordinates generated from different experiments that involve the same participants, as recommended (Turkeltaub et al., 2012). We restricted studies to those reporting on the instant effects of performing a motor task in the mirror therapy paradigm to ensure homogeneity. As a result only 14 experiments (with 152 foci) met the inclusion for the meta-analysis, which is less than the number of experiments (17–20) recommended to perform an ALE meta-analysis (Eickhoff et al., 2016). This would have lowered the power of part of the analyses and readers should interpret the results with caution. Among the experiments included in the analyses, the instructions given to the participants appeared to be rather brief which were to observe movements of the inverted virtual or mirrored hand. As a consequence, there could have been variability among the participants in how observations were made such as on the entire or part of the hand. Another variability identified was in the number of hands displayed for observation by the participants such as unilateral or bilateral hands. Previous studies revealed activations in the visual and motor cortices were modulated by participants' observing one versus two limbs (Hadoush et al., 2013; Deconinck et al., 2015). These variabilities could have confounded the results and is regarded as a limitation of the study.

## Conclusion

Neural substrates that subserve the mental processes of MVI could only be traced from individual neuroimaging studies and reviews with qualitative approaches. The present study provided the first quantitative account on neural substrates that are found collectively associated with the neural processes underlying MVI. The findings from this study showed the effect of MVI in facilitating neural activities in the diverse regions of the human brain, including the fronto-temporo-parietal, occipital and cerebellar regions. The lateralization of basic visual processing toward the ipsilateral hemisphere is indicative that MVI can potentially influence the neural system.

## Author Contributions

UB: conceptualization, methodology, formal analysis, data curation, writing—original draft, and visualization. GK: methodology, validation, and writing—review and editing. SW: conceptualization, methodology, resources, writing—review and editing, and supervision. CC: conceptualization, methodology, writing—review and editing, and supervision. All authors: contributed to the article and approved the submitted version.

## Conflict of Interest

The authors declare that the research was conducted in the absence of any commercial or financial relationships that could be construed as a potential conflict of interest.
