# Supplementary material for: Neural Processes Underlying Mirror-Induced Visual Illusion: An Activation Likelihood Estimation Meta-Analysis
Source: Front Hum Neurosci. 2020 Jul 31;14:276. doi: 10.3389/fnhum.2020.00276 (PMC7412952; doi:10.3389/fnhum.2020.00276)
Supplement: Supplementary file 1 [file Table_1.DOCX]

**Appendices**

**Appendix A:** Overall meta-analytic results showing brain areas consistently found to be associated with the MVI condition when contrasted with the rest/baseline condition (13 experiments with 152 foci). Note: The MVI tasks involved moving left hand (right-left flipped coordinates for moving right hand)

| Cluster Anatomical location Side BA Z-score ALE MNI Coordinates  value X Y Z | | | | | | | | |
| --- | --- | --- | --- | --- | --- | --- | --- | --- |
| 1 | Primary motor cortex | R | 4 | 7.63 | 0.033 | 38 | -20 | 58 |
|  | Inferior parietal lobule | R | 40 | 3.32 | 0.009 | 38 | -36 | 58 |
| 2 | Premotor cortex | R | 6 | 6.12 | 0.024 | 2 | -6 | 60 |
|  | Primary motor cortex | L | 4 | 5.15 | 0.019 | -40 | -10 | 56 |
|  | Primary somatosensory cortex | L | 3 | 4.70 | 0.016 | -56 | -12 | 40 |
|  | Premotor cortex | L | 6 | 4.34 | 0.014 | -28 | -8 | 56 |
|  | Superior frontal gyrus | L | 6 | 3.61 | 0.011 | -12 | -6 | 66 |
| 3 | Superior parietal lobule | L | 7 | 4.10 | 0.013 | -30 | -52 | 58 |
|  | Inferior parietal lobule | L | 40 | 3.45 | 0.010 | -40 | -36 | 52 |
|  | Precuneus | L | 7 | 2.98 | 0.001 | -18 | -60 | 58 |
| 4 | Cerebellum (anterior lobe) | L | - | 5.60 | 0.021 | -6 | -58 | -10 |
|  | Cerebellum (posterior lobe) | L | - | 3.26 | 0.009 | -6 | -66 | -24 |

*Abbreviations:* ALE, Activation Likelihood Estimation; BA, Brodmann’s Area; MNI, Montreal neurological institute.

**Appendix B:** ALE meta-analytic results showing brain areas consistently found as a result of contrast between MVI condition with active hand movement without mirror visual feedback (30 foci). Note: The MVI tasks involved movements of the left hand (right-left flipped coordinates for right hand movements)

| Cluster Anatomical location Side BA Z-score ALE MNI Coordinates  value X Y Z | | | | | | | | |
| --- | --- | --- | --- | --- | --- | --- | --- | --- |
| 1 | Cuneus | L | 19 | 4.69 | 0.012 | -16 | -84 | 38 |
| 1 | Lingual gyrus | L | - | 4.50 | 0.011 | -18 | -78 | 2 |
| 1 | Middle occipital gyrus (V2 visual area) | L | 18 | 4.16 | 0.009 | -26 | -88 | 12 |
| 1 | Superior temporal gyrus | L | 22 | 3.95 | 0.008 | -52 | -48 | 14 |
| 1 | Middle temporal (fusiform) gyrus | L | 37 | 2.87 | 0.005 | -46 | -64 | 2 |
| 1 | Precuneus | L | 19 | 2.87 | 0.005 | -10 | -78 | 48 |
| 1 | Cerebellum (posterior lobe) | L | - | 2.87 | 0.005 | -20 | -76 | -12 |

*Abbreviations:* ALE, Activation Likelihood Estimation; BA, Brodmann’s Area; MNI, Montreal neurological institute.
